# A20 deubiquitinase controls PGC-1α expression in the adipose tissue

**DOI:** 10.1186/s12944-018-0740-6

**Published:** 2018-04-20

**Authors:** Bruna Bombassaro, Leticia M. Ignacio-Souza, Carla E. Nunez, Daniela S. Razolli, Rafael M. Pedro, Andressa Coope, Eliana P. Araujo, Elinton A. Chaim, Licio A. Velloso

**Affiliations:** 1 Laboratory of Cell Signaling, Obesity and Comorbidities Research Center University of Campinas, Campinas, Brazil; 2 Department of Surgery, University of Campinas, Campinas, Brazil; 3 Laboratory of Cell Signaling, Faculdade de Ciencias Medicas da Universidade Estadual de Campinas, Campinas, SP 13084 970 Brazil

**Keywords:** Fat, Ubiquitination, Glucose, Diet, Obesity

## Abstract

**Background:**

Peroxisome proliferator-activated receptor γ coactivator- 1alpha (PGC-1α) plays an important role in whole body metabolism and, particularly in glucose homeostasis. Its expression is highly regulated and, small variations in tissue levels can have a major impact in a number of physiological and pathological conditions. Recent studies have shown that the ubiquitin/proteasome system plays a role in the control of PGC-1α degradation.

**Methods:**

Here we evaluated the interaction of PGC-1α with the protein A20, which plays a dual-role in the control of the ubiquitin/proteasome system acting as a deubiquitinase and as an E3 ligase. We employed immunoprecipitation, quantitative real-time PCR and immunofluorescence staining to evaluate PGC-1α, A20, PPARγ and ubiquitin in the adipose tissue of humans and mice.

**Results:**

In distinct sites of the adipose tissue, A20 binds to PGC-1α. At least in the subcutaneous fat of humans and mice the levels of PGC-1α decrease during obesity, while its physical association with A20 increases. The inhibition of A20 leads to a reduction of PGC-1α and PPARγ expression, suggesting that A20 acts as a protective factor against PGC-1α disposal.

**Conclusion:**

We provide evidence that mechanisms regulating PGC-1α ubiquitination are potentially involved in the control of the function of this transcriptional co-activator.

## Background

Peroxisome proliferator-activated receptor γ coactivator 1 alpha (PGC-1α) is a co-activator of transcription involved in the control of a number of metabolic functions in a diversity of tissues in mammals [1, 2]. It was first identified in brown adipose tissue (BAT) due to its important role in the modulation of peroxisome proliferator-activated Receptor gamma (PPARγ) – dependent uncoupling protein-1 (UCP1) expression [3, 4]. Because of its pleotropic functions, the regulation of PGC-1α expression by extracellular signals is complex and varies in distinct tissues. Thus, for example, in BAT and pancreatic islets it is induced by cold [3–6], in skeletal muscle it is induced by exercise [7, 8], and in the liver it is induced by fasting [9]. In addition, at the intracellular level, PGC-1α gene expression can be controlled by many different mechanisms, such as cyclic AMP and Ca^+ 2^ signaling through cyclic AMP response element-binding protein (CREB) [10, 11], NO-induced cyclic GMP pathway [12], myocyte enhancer factor (MEF), and even through an auto-regulatory loop in white adipose tissue (WAT) leading to PPARγ activation [13].

The availability of PGC-1α in a given cell impacts on the immediate regulation of target gene transcription, which means that both production, i.e., transcription and translation, and degradation of PGC-1α must be tightly controlled [1]. If in one hand the control of PGC-1α production has been widely studied, on the other hand, little is known about the processes and mechanisms that lead to its degradation. Under physiological conditions the half-life of PGC-1α is as short as 2–3 h [14]. However, this can be modulated by different intracellular signals such as p38 mitogen-activated protein kinases (MAPK) and p160 myb [14, 15]. Also, recent studies have shown that PGC-1α is degraded through the ubiquitin-proteasome system [16, 17].

The ubiquitination of target proteins plays an important and wide-ranging role in the homeostasis of cells. Typically it targets damaged proteins to proteasome degradation [18, 19] and potentially detrimental protein aggregates that cannot be degraded by the proteasome, to autophagy [20, 21]. There are a number of steps and mechanisms involved in the activation and control of the ubiquitination machinery [18]. One of such regulatory events is carried out by a family of proteins with deubiquitinase activity [18]. Interestingly, deubiquitination of target proteins plays an important role in the control of inflammatory signaling pathways, particularly by the action of A20 deubiquitinase through the regulation of nuclear factor-kappaB (NFκB) activity [21, 22].

In obesity, the hypertrophic WAT is targeted by a low-grade inflammatory process on which the activation of signaling through NFκB plays an important role [23]. An important outcome of this inflammatory process is the induction of insulin resistance and disruption of metabolic pathways [23]. Defective signaling though PPARγ plays an important role in adipose tissue insulin resistance and, the use of thiazolidinediones can greatly improve this phenotype [24]. It has been shown that PGC-1α expression is greatly reduced in WAT of obese subjects. Therefore, we hypothesized that changes in ubiquitination, potentially involving the activity of A20, could explain at least part of the changes in WAT PGC-1α expression. To test this hypothesis we evaluated PGC-1α and A20 expression and PGC-1α ubiquitination in the WAT of obese humans before and after body mass reduction resulting from bariatric surgery. In addition, we evaluated the same proteins and the effect of an oligonucleotide antisense (ASO) inhibition of A20 in the adipose tissue of an animal model of obesity. As a whole, our results show that PGC-1α expression is reduced in obesity and its association with A20 is a protective factor against this reduction. When the expression of A20 is reduced by ASO the levels of PGC-1α decrease and the animals become glucose intolerant.

### Methods

#### Experimental animals

Six-week old male Swiss mice were fed on standard rodent chow or on a high-fat diet for 16 weeks. The macronutrient composition of diets is presented in Table 1. All experimental procedures were performed in accordance with the guidelines of the Brazilian College for Animal Experimentation and were approved by the University of Campinas Ethics Committee (#CEUA 2216–1).

**Table 1:** Composition of the diets

	Diets	
	Chow	High-fat
Protein (g%)	22.5	26
Fat (g%)	4.5	35
Carbohydrate (g%)	55	26
Fiber (g%)	8	6
Ash (g%)	10	7
Total	100	100
Energy value (kCal/g)	3.5	5.2

#### Human adipose tissue

Subcutaneous adipose tissue from the abdominal wall was collected from 12 obese subjects during a Roux-in-Y gastric bypass surgery at the Clinics Hospital of the University of Campinas. The adipose tissue from nine lean volunteers was collected at the Laboratory of Investigation in Metabolism and Diabetes, University of Campinas. The exclusion criteria were, as follows: inflammatory or infectious disease acute or chronic, neurological disease, psychiatric illness, smoking, alcohol consumption greater than or equal to 30 g per day for men and 15 g per day for women, use of illicit drug, use of NSAIDs or corticosteroids, cancer, pregnancy, liver enzyme levels > 3-times the upper limit of normal, chronic renal failure. Patients were informed about the project and signed a consent form. Volunteers could, at any time, withdraw from the project. The study was evaluated and approved by the University of Campinas Ethics Committee for Medical Research (#833/2010).

#### Antisense oligonucleotide (ASO) treatment

An antisense oligonucleotide (ASO) targeting TNFAIP3 (gene coding for the A20 protein) and a scrambled ASO were designed and used to treat the experimental animals. The sequence of the TNFAIP3 ASO was 5’ACCCCAGTATTTGATCTTGT 3′ and the scramble ASO was 5’TACTACGCGCATTCTTATTG 3′ (Invitrogen, São Paulo, Brazil). The lyophilized ASOs were suspended in Tris-acetate-EDTA Buffer (40 mM Tris, 20 mM acetic acid, 1 mM EDTA) at 1 nmol/μL and diluted in saline for intraperitoneal injection, once a day for 7 days. This method was adapted from a previously published study [25].

#### Intraperitoneal glucose tolerance test (ipGTT)

Glucose was determined in blood using a glucometer from Abbott (Opptimum, Abbott Diabetes Care, Inc., Alameda, CA, USA). After an overnight fasting, the animals were fed during 1 h and then fasted for 4 h. After the collection of the first blood sample (time 0), 20% glucose (2.0 g/kg body weight) was administered via intraperitoneal injection. Blood samples from tails were collected at different times for the determination of glucose concentration.

#### Immunoblotting

The adipose tissue specimens were homogenized in a tissue homogenizer (Polytron-Aggregate, Kinematica, Littau/Luzern, Switzerland) at maximum speed in an anti-protease cocktail (10 mmol/L imidazole, pH 8.0, 4 mmol/L EDTA, 1 mmol/L, aprotinin, 2.5 mg/L leupeptin, 30 mg/L trypsin inhibitor, 200 μmol/L DTT and 200 μmol/L phenylmethylsulfonyl fluoride). After sonication, an aliquot of the extracts was collected and the total protein content was determined by the dye-binding protein assay kit (Bio-Rad Laboratories, Hercules, CA). Samples containing 100 μg of protein were incubated for 5 min at 95 °C with 4× concentrated Laemmli sample buffer (1 mmol sodium phosphate/L, pH 7.8, 0.1% bromophenol blue, 50% glycerol, 10% SDS, 2% mercaptoethanol) or immunoprecipitated (500 μg of protein) with anti-A20 or anti-PGC-1α (4:1, vol/vol) and then run on 10% polyacrylamide gels during approximately 4 h. The amounts of antibodies used in the immunoprecipitation assays were sufficient to immunodeplete the samples as evaluated by running an immunoblotting assay of the supernatants. Electrotransfer of proteins to nitrocellulose membranes (Bio-Rad) was performed in a Trans Blot SD Semi-Dry Transfer Cell (Bio-Rad) for 1 h at 15 V (constant) in buffer containing methanol and SDS. After transfer, the membranes were blocked with 5% skimmed milk in Tween−/Tris-buffered saline (TTBS) (10 mmolTris/L, 150 mmolNaCl/L, and 0.5% Tween 20) overnight at 4 °C. A20, PGC-1α and ubiquitin were detected in the membranes after overnight incubation at 4 °C with primary antibodies (A20, sc166692; PGC-1α, sc13067, from Santa Cruz Biotechnology, Santa Cruz, CA and ubiquitin, ab7780 from AbCam, Cambridge, MA, USA; diluted 1:500 in TTBS containing 3% dry skimmed milk). The membranes were then incubated with a secondary specific immunoglobulin G antibody (diluted 1:5000 in TTBS containing 3% dry skimmed milk) for 2 h at room temperature. Enhanced chemiluminescence (SuperSignal West Pico; Pierce) after incubation with a horseradish peroxidase–conjugated secondary antibody was used for detection by autoradiography. Band intensities were quantified by optical densitometry (UN-Scan-it Gel 6.1, Orem, Utah, USA).

#### RNA extraction and real-time-qPCR

The samples were homogenized in TRIzol reagent (Invitrogen, São Paulo, Brasil) in a tissue homogenizer (Polytron-Agregate, Kinematica, Littau/Luzern, Switzerland) at maximum speed. The total RNA content was then isolated according to the manufacturer’s instructions, quantified and analyzed by spectrophotometry (NanoDrop 8000, Thermo Scientific, Wilmington, DE, USA). The integrity of RNA and the total amount were assessed by Nanodrop (Nanodrp 8000 Thermo Scientific). cDNA synthesis was performed in 3 μg of total RNA, according the manufacturer’s instructions (High Capacity cDNA Reverse Transcription Kit, Life Technologies, Van Allen Way Carlsbad, CA, USA). The TaqMan System was used in association with real-time PCR to detect A20 (TNFAIP3), PGC-1α and PPARγ in the brown, visceral and subcutaneous adipose tissue (Mm00437121_m1; Mm44718 3 m1; Mm01184322_m1, respectively - Life Technologies, Van Allen Way Carlsbad, CA, USA) and the mouse GAPDH gene was used as an endogenous control (#4352339E).

#### Immunofluorescence staining

For histological evaluation, brown and white adipose tissue samples were fixed in paraformaldehyde (4% final concentration in phosphate-buffered saline [PBS; 50 mmol/L of NaH2PO4 · H2O; 5 mmol/L of KCl; 1.5 mmol/L of MgCl2 · 6H2O; and 80.1 mmol/L of NaCl; pH 7.4]) and processed routinely for embedding in a paraffin block. The samples were submitted to dehydration (alcohol at 70%, 80%, 90%, 95%, and absolute alcohol) being diaphanized by immersion in xylol and embedded in paraffin. Subsequently, the hydrated (alcohol at absolute, 95, 90, 80, and 70% concentrations) 5.0 um paraffin sections were processed for immunofluorescence staining using the Ubiquitin, A20 and PGC-1α antibodies (sc271289; sc166692, sc13067 respectively - Santa Cruz Biotechnology, Santa Cruz, CA) and the secondary antibodies conjugated to FITC or rhodamine (sc2777; sc2092, respectively - Santa Cruz Biotechnology, Santa Cruz, CA). The images were obtained using a Confocal Laser Microscopy (LSM510, Zeiss, New York, NY). Analyses of results were performed using a Leica Application Suite V3.6 (Switzerland).

#### Statistical analysis

Results are presented as means ± SE. The homogeneity of variances was checked by Levene’s test to verify if the data were parametric. When necessary, to correct for variance heterogeneity or non-normality, data were log-transformed [26]. The results were analyzed by Student-test or One-way ANOVA and complemented by the Tukey test to determine the significance of individual differences. The level of significance was set at *p* < 0.05. The data were analyzed using Statistic for Windows, 7.0 (StatSoft, Inc., Tulsa, OK, USA).

## Results

### PGC-1α and A20 are reduced in the adipose tissue of obese subjects

**Fig. 1:**
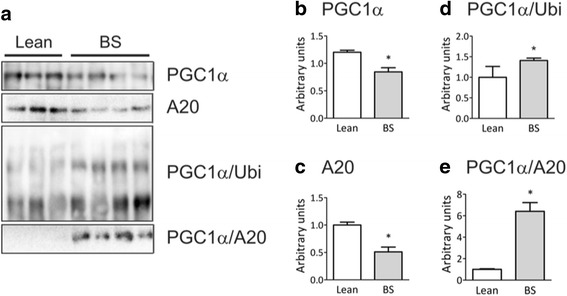
PGC-1α and A20 expression in the adipose tissue of humans. Samples containing 500 μg total protein from abdominal subcutaneous adipose tissue specimens, collected from 9 lean volunteers and 12 obese subjects during a Roux-in-Y gastric bypass (BS) were used in immunoprecipitation experiments employing anti-PGC-1α (**a**, **b**, **d** and **e**) or anti-A20 (**a** and **c**) as primary antibodies. The immunoprecipitation procedure used antibodies sufficient to immunodeplete the sample. The immunoprecipitates were separated by SDS-PAGE, transferred to nitrocellulose membranes and blotted with anti-PGC-1α (**a** and **b**), anti-A20 (**a**, **c** and **e**) or anti-ubiquitin (**a** and **d**) antibodies. In A, typical blots are depicted; in B-E the quantification of bands is graphically represented. *p < 0.05 vs. lean

Except for body mass index, the groups of subjects presented no differences in age, glucose, HbA1c and cholesterol (Table 2). Nevertheless, the protein expressions of both PGC-1α and A20 were significantly reduced as compared to control (Fig. 1a-c), while the associations of PGC-1α with ubiquitin and A20 were increased (Fig. 1a, d and e).

**Table 2:** Anthropometric and biochemical parameters of subjects

Parameters	Lean	Obese
Number	9	12
Gender (F/M)	8/1	8/4
Age	32 ± 4	38 ± 6
BMI (kg/m^2^)	22 ± 2	36 ± 3*
Glucose (mg/dL)	77 ± 7	80 ± 7
HbA1c (%)	5.3 ± 0.4	5.4 ± 0.3
Total Cholesterol (mg/dL)	193 ± 29	165 ± 17

Data are presented as means ± standard deviation

*BMI* body mass index, *HbA1c* glycated hemoglobin

### PGC-1α and A20 are differentially expressed in distinct sites and types of adipose tissue in mice

**Fig. 2:**
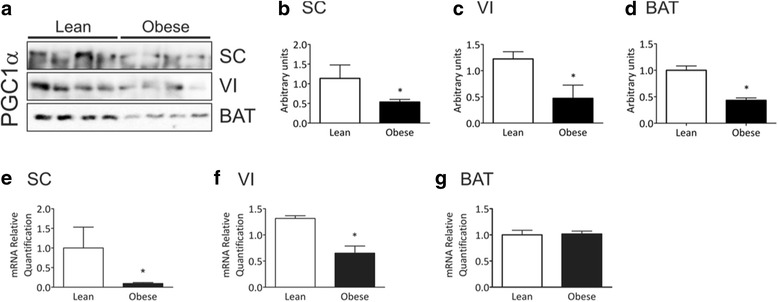
PGC-1α expression in distinct adipose tissue depots of mice. Samples containing 500 μg total protein (**a-d**) or 25 ng cDNA (E-G) from subcutaneous (SC), visceral (VI) or brown (BAT) adipose tissue depots from lean or obese mice were used in immunoprecipitation (**a-d**) or quantitative real-time PCR (**e-g**) experiments. In **a-d**, samples were immunoprecipitated employing anti-PGC-1α as primary antibody. The immunoprecipitation procedure used antibodies sufficient to immunodeplete the sample. The immunoprecipitates were separated by SDS-PAGE, transferred to nitrocellulose membranes and blotted with anti-PGC-1α antibody. In **a**, typical blots are depicted; in **b-d** the quantification of bands is graphically represented. In **e-g**, the quantification of PGC-1α mRNA obtained by quantitative real-time PCR is represented graphically. In all experiments *n* = 5–6, *p < 0.05 vs. lean

**Fig. 3:**
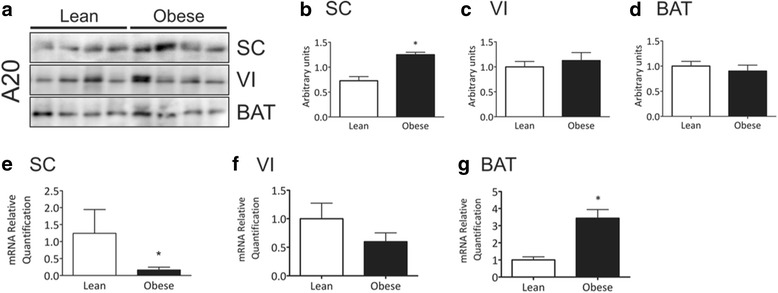
A20 expression in distinct adipose tissue depots of mice. Samples containing 500 μg total protein (**a-d**) or 25 ng cDNA (**e-g**) from subcutaneous (SC), visceral (VI) or brown (BAT) adipose tissue depots from lean or obese mice were used in immunoprecipitation (**a-d**) or quantitative real-time PCR (**e-g**) experiments. In **a-d**, samples were immunoprecipitated employing anti-A20 as primary antibody. The immunoprecipitation procedure used antibodies sufficient to immunodeplete the sample. The immunoprecipitates were separated by SDS-PAGE, transferred to nitrocellulose membranes and blotted with anti-A20 antibody. In **a**, typical blots are depicted; in **b-d** the quantification of bands is graphically represented. In **e-g**, the quantification of A20 mRNA obtained by quantitative real-time PCR is represented graphically. In all experiments *n* = 6, *p < 0.05 vs. lean

**Fig. 4:**
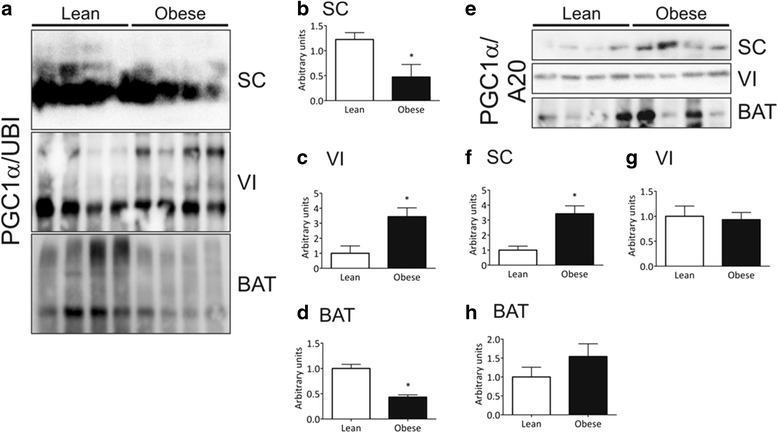
PGC-1α association with ubiquitin and A20 in distinct adipose tissue depots of mice. Samples containing 500 μg total protein from subcutaneous (SC), visceral (VI) or brown (BAT) adipose tissue depots from lean or obese mice were used in immunoprecipitation experiments employing anti-PGC-1α as primary antibody. The immunoprecipitation procedure used antibodies sufficient to immunodeplete the sample. The immunoprecipitates were separated by SDS-PAGE, transferred to nitrocellulose membranes and blotted with anti-ubiquitin **(a-d**) or anti-A20 (**e-h**) antibodies. In **a** and **e**, typical blots are depicted; in **b-d** and **f-h** the quantification of bands is graphically represented. In all experiments *n* = 5–6, *p < 0.05 vs. lean

Because of the differences in function and potential for inflammatory response presented by the distinct sites and types of adipose tissue, we evaluated the expressions of PGC-1α and A20 in samples obtained from the brown adipose tissue and from either visceral or subcutaneous fat from lean and obese mice. As depicted in Fig. 2a-d, PGC-1α protein was consistently reduced in all types and sites of adipose tissue of obese mice. The mRNA expression of PGC-1α was reduced in both visceral and subcutaneous fat (Fig. 2e and f) but was unchanged in the brown adipose tissue (Fig. 2g) of obese mice. Differently of obese humans, obese mice presented increased expression of A20 protein only in the subcutaneous fat depot, while in visceral and brown adipose tissue the expression of A20 was similar between lean and obese animals (Fig. 3a-d). Regarding mRNA levels, A20 expression in obese mice was unchanged in visceral fat, reduced in subcutaneous fat and increased in the brown adipose tissue (Fig. 3e-g). The association of PGC-1α with ubiquitin was differently regulated when comparing the distinct types and sites of adipose tissue, while in subcutaneous fat and brown adipose tissue the association was reduced, in visceral fat it was increased (Fig. 4a-d). The association of PGC-1α with A20 was also differentially regulated among the distinct types and sites of adipose tissue. Thus, it was increased in the subcutaneous fat, while unchanged in visceral fat and brown adipose tissue (Fig. 4e-h).

### Evaluation of PGC-1α co-expression with ubiquitin and A20 by immunofluorescence staining

**Fig. 5:**
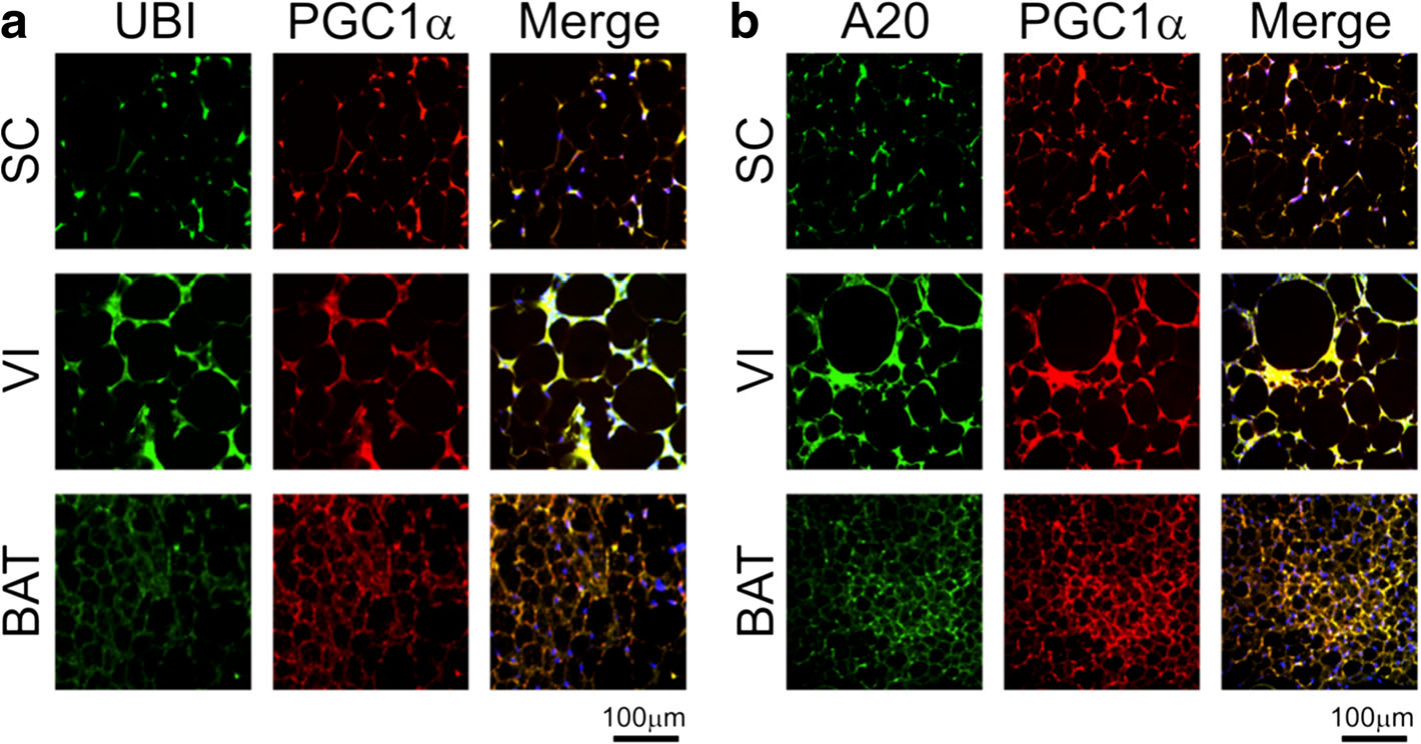
Immunofluoescence evaluation of the co-localizations of PGC-1α with ubiquitin and A20. Subcutaneous (SC), visceral (VI) or brown (BAT) adipose tissue specimens from obese mice were used in immunofluorescence assays to detect the presence and co-localizations of PGC-1α and ubiquitin (Ubi) (**a**) and PGC-1α and A20 (**b**). Figures are typical representations obtained from a total of two distinct experiments

The changes in association between PGC-1α and ubiquitin or A20 detected by the co-immunoprecipitation assays were mostly confirmed by immunofluorescence staining. Thus, in obese mice there was a clear increase in the co-localization of PGC-1α with ubiquitin in visceral fat (Fig. 5a), while reductions were detected in brown adipose tissue and subcutaneous fat (Fig. 5a). The association of PGC-1α with A20 was increased in subcutaneous fat while no major changes were detected in brown adipose tissue and visceral fat (Fig. 5b).

### Inhibition of A20 impairs whole body glucose homeostasis

**Fig. 6:**
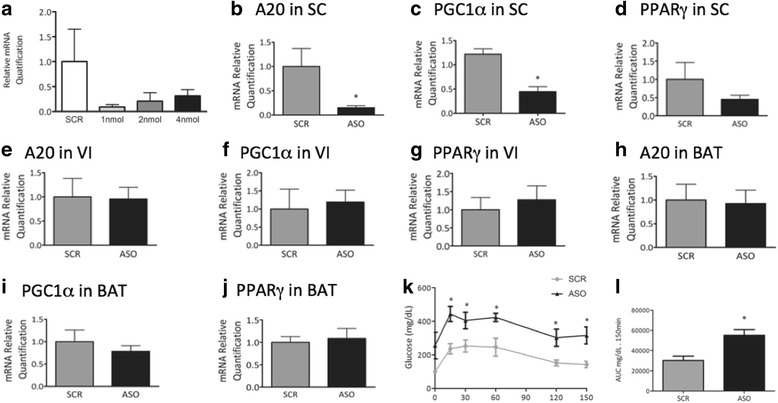
Inhibiting A20 expression. Obese Swiss mice were treated once a day, for 7 days, with a single intraperitoneal injection of a 100 μl solution containing either A20 antisense (ASO) or scrambled (SCR) oligonucleotide. Fragments from subcutaneous (SC) (**a-d**), visceral (VI) (**e-g**) or brown (BAT) (**h-j**) adipose tissue were employed in quantitative real-time PCR experiments. In **a**, A20 transcript expression was evaluated in a dose-response experiment to determine the efficiency of the method. In **b-l**, the doses of ASO and SCR employed were always 2 nmol in 100 μl/dose. In **b-j**, the expressions of A20 (**b**, **e**, **h**); PGC-1α (**c**, **f**, **i**); and PPARγ (**d**, **g**, **j**) were determined and are represented graphically. The blood glucose levels during a glucose tolerance test (**k**) and the respective area under the glucose curve (**l**) are represented graphically. In all experiments *n* = 8; *p < 0.05 vs. SCR

To test the hypothesis that A20 controls the levels of PGC-1α which could potentially impact on the control of glucose homeostasis, we treated obese mice with an antisense oligonucleotide (ASO) against A20 and evaluated the expression of related proteins and whole body energy homeostasis. A dose-response experiment showed that the treatment with 1.0, 2.0 or 4.0 nmol ASO per day for 7 days were sufficient to reduce the expression of either A20 or PGC-1α in the subcutaneous adipose tissue (Fig. 6a and b). The dose of 2.0 nmol/day was used in the remaining experiments. The intraperitoneal treatment with the A20 ASO resulted in significant reduction of A20 only in the subcutaneous fat (Fig. 6b). In brown adipose tissue and visceral fat, no changes in A20 levels were detected following A20 ASO treatment (Fig. 6e and h). The expressions of PGC-1α and PPARγ were reduced only in the subcutaneous fat (Fig. 6c and d), while no changes were detected in brown adipose tissue and visceral fat (Fig. 6f/i and g/j). The inhibition of A20 resulted in no changes in food intake and body mass (not shown). However, fasting glucose levels (Fig. 6k) and the area under glucose curve during a glucose tolerance test were severely worsened by the treatment with the A20 ASO (Fig. 6l).

## Discussion

In this study we demonstrate that PGC-1α associates with A20 in the adipose tissue of humans and mice. This association is independently regulated in distinct types and sites of the adipose tissue, and increases in obesity. In all conditions analyzed in both humans and mice, the expression of adipose tissue PGC-1α was reduced during obesity. In most conditions, particularly in the subcutaneous fat, the expression of PGC-1α was inversely correlated with its physical association with A20, thus, raising the question of whether A20 acts to protect or to enhance PGC-1α degradation.

A20 is a zinc finger protein identified as a regulator of NFκB activity in response to TNFα [27]. It has a dual role, acting as a deubiquitinase due to the catalytic activity of its N-terminal region; and, as an E3 ligase due to its C-terminal region containing the zing finger domain [28, 29]. The regulation of NFκB activity is the most extensively studied function of A20. It is currently known that upon TNFα signal transduction through TNFR1, A20 removes the polyubiquitinated lysine-63 residues of receptor-interacting protein-1 (RIP1) preventing its interaction with NEMO [30]. In addition, A20 adds a lysine-48 polyubiquitinated tail to RIP1, triggering its proteasome-mediated degradation [31]. These combined catalytic actions result in the inhibition of NFκB activity, and thus, attenuation of inflammation. Therefore, A20 exerts an important function in the control of NFκB-dependent inflammatory signaling.

Subclinical inflammation is a common feature of human and experimental obesity [23, 32]. Activation of TLR4 signal transduction and induction of endoplasmic reticulum stress (ERS), are regarded as two of the most important mechanisms triggering the inflammatory activity related to obesity [23, 32–34]. Once active, both TLR4 signaling and ERS induce intracellular inflammatory signal transduction through at least two important signaling intermediaries, JNK and NFκB [35–37]. As a consequence of the activation of inflammatory regulated serine-threonine kinases such as JNK, IKK the insulin receptor and some of its important primary substrates are negatively modulated by serine phosphorylation resulting in the impairment of the insulin signal transduction [37, 38]. There are a number of cell specific outcomes resulting from inflammation-induced insulin resistance. In the adipose tissue, one such outcome is the defective activation of PPARγ which leads to an anomalous distribution of fat and increased expression of inflammatory adipokines, further enhancing inflammation and insulin resistance; thus, creating a vicious cycle [24, 39].

Because PGC-1α is an important regulator of PPARγ expression we asked whether in obesity the expression of PGC-1α would be reduced, and, if so, what would be the role of A20 in this context. First, we showed that in both obese humans and mice the expression of PGC-1α was indeed reduced. In the case of mice, this was occurring in visceral and subcutaneous fat and also in BAT. These findings confirm previous studies that evaluated PGC-1α in different groups of obese subjects and showed a consistent reduction in both visceral and subcutaneous depots [40–42].

Next, to test our working hypothesis, we determined the levels of A20 and its interaction with PGC-1α. In both humans and mice, in the subcutaneous fat, A20/PGC-1α association was increased, while in mice visceral fat and BAT it was unchanged. No previous study has evaluated the association of PGC-1α with A20. However, in other studies, the activity of A20 related to its ability to bind to distinct proteins resulted, in most times, in the modification of the target protein expression levels [28, 30, 31, 43].

A20 can act to modify a given target protein expression by at least two mechanisms, deubiquitination and/or E3 ligase activity [28, 30]. Although we have not evaluated the potential involvement of the distinct functions of A20 to control PGC-1α expression, we employed a method to reduce A20 expression and looked for the outcome on PGC-1α levels and modulation of metabolic functions. Interestingly, the use of an ASO targeting A20 resulted in the reduction of A20 expression only in the subcutaneous fat depot, while no changes were detected in the visceral fat and BAT. The reason for this apparent specificity was not investigated but it is currently known that mRNA targeting with ASOs or even siRNA approaches can result in reduction, no modulation or even increase of the target expression [44–46]. This can be due to factors such as, for example, differences in the uptake of the oligonucleotide or the presence of endogenous siRNAs.

In the subcutaneous adipose tissue, the reduction of A20 was accompanied by a reduction of PGC-1α and PPARγ, while in the other two adipose sites no changes in the expression of PGC-1α and PPARγ were detected. As an outcome of A20 inhibition there was an increase in fasting glucose levels and in the area under glucose curve during a glucose tolerance test, which were independent on changes in caloric intake and body mass. In a number of different contexts the reductions in the expression of either PGC-1α or PPARγ in the adipose tissue have been shown to result in impaired glucose tolerance [47–49]. Thus, the role of A20 controlling subcutaneous adipose tissue levels of PGC-1α impacts on PPARγ levels resulting in an important change in whole body glucose homeostasis.

## Conclusion

This study has shown that in distinct sites of the adipose tissue A20 binds to PGC-1α. At least in the subcutaneous fat of humans and mice the levels of PGC-1α decrease during obesity, while its physical association with A20 increases. The inhibition of A20 in this site produces a reduction of PGC-1α and PPARγ expression, suggesting that A20 acts as a protective factor against PGC-1α disposal. Thus, we provide additional [16] evidence that mechanisms regulating PGC-1α ubiquitination are potentially involved in the control of the function of this transcriptional co-activator.

## Abbreviations

BAT: Brown adipose tissue
CREB: cAMP Response Element-Binding protein
IKK: IκB Kinase
JNK: c-Jun N-terminal Kinase
MAPK: Mitogen-activated protein kinases
MEF: Myocyte enhancer factor
NFκB: Nuclear Factor-KappaB
PGC1α: Peroxisome proliferator-activated receptor gamma coactivator 1-alpha
PPARγ: Peroxisome proliferator-activated receptor gamma
RIP-1: Receptor-interacting protein-1
TNFα: Tumor necrosis factor alpha
UCP1: Uncoupling protein-1
WAT: White adipose tissue
